# Metagenomic sequencing reveals time, host, and body compartment-specific viral dynamics after lung transplantation

**DOI:** 10.1186/s40168-022-01244-9

**Published:** 2022-04-23

**Authors:** Stefanie Widder, Irene Görzer, Benjamin Friedel, Nina Rahimi, Stefan Schwarz, Peter Jaksch, Sylvia Knapp, Elisabeth Puchhammer-Stöckl

**Affiliations:** 1 Research Laboratory of Infection Biology, Department of Medicine I, Medical University of Vienna, Vienna, Austria; 2 Konrad Lorenz Institute for Evolution and Cognition Research, Klosterneuburg, Austria; 3 Center of Virology, Medical University Vienna, Vienna, Austria; 4 Department for Internal Medicine, Diabetology, Endocrinology, Diakonissenkrankenhaus, ViDia Kliniken, Karlsruhe, Germany; 5 Division of Thoracic Surgery, Department of Surgery, Medical University of Vienna, Vienna, Austria

**Keywords:** Virus metagenomics, Network analysis, Viral community clusters, Lung transplantation, Data modeling

## Abstract

**Background:**

The virome of lung transplant recipients (LTRs) under immunosuppressive therapy is dominated by non-pathogenic *Anelloviridae* and further includes several pathogenic viruses such as Herpesviruses or respiratory viruses. It is unclear whether the donor-derived virome in the transplanted lung influences recipient virome dynamics in other body compartments and if so, to which degree. Likewise, it is unknown whether dependencies exist among virus populations that mutually shape viral loads and kinetics.

**Results:**

To address these questions, we characterized viral communities in airways and plasma of 49 LTRs and analyzed their abundance patterns in a data modeling approach. We found distinct viral clusters that were specific for body compartments and displayed independent dynamics. These clusters robustly gathered specific viral species across the patient cohort. In the lung, viral cluster abundance associated with time after transplantation and we detected mutual exclusion of viral species within the same human host. In plasma, viral cluster dynamics were associated with the indication for transplantation lacking significant short-time changes. Interestingly, pathogenic viruses in the plasma co-occurred specifically with *Alpha torque virus* genogroup 4 and *Gamma torque virus* strains suggesting shared functional or ecological requirements.

**Conclusions:**

In summary, the detailed analysis of virome dynamics after lung transplantation revealed host, body compartment, and time-specific dependency patterns among viruses. Furthermore, our results suggested genetic adaptation to the host microenvironment at the level of the virome and support the hypothesis of functional complementarity between *Anellovirus* groups and other persistent viruses.

**Video abstract**

**Supplementary Information:**

The online version contains supplementary material available at 10.1186/s40168-022-01244-9.

## Introduction

Within the last years, it was shown that a multitude of different eukaryotic viruses colonize the human host [1, 2]. This eukaryotic human virome may vary over time, and differs between healthy persons and specific patient populations as well as between different human body sites as blood or lung [3, 4]. In immunosuppressed patients, as solid organ transplant recipients, who undergo lifelong drug induced decrease of different immune responses, the level of distinct virus populations is massively increased [4–6]. While in a number of pathogenic viruses, as herpesviruses or polyomaviruses, replication under these circumstances may cause disease, the most prominent eukaryotic virus populations found in clinical material of transplant recipients belong to the non-pathogenic *Anelloviridae*, consisting of *Alpha*-, *Beta*-, and *Gamma*-*torque viruses* [4, 5, 7, 8]. Within the last years, it turned out that among these, especially the group of *Alpha torque viruses* (TTV) are strongly elevated in patients under immunosuppressive therapy after solid organ transplantation [9, 10]. The overall total level of TTV, which comprise 5 genogroups (TTV1-5), and numerous TTV strains, was shown meanwhile to serve as a marker for the level of immunosuppression especially after lung or kidney transplantation [9, 11, 12]. Previous data in lung transplant recipients have provided evidence that there is not only a multitude of different TTV strains detected within a single patient at a given time point in BAL or blood, but that TTV populations also change substantially over time within a single patient [4].

It is known that a variety of different viruses may co-exist within the same human compartment [5]. While it is well established that the human bacteria microbiome engages in a plethora of interactions, it is unknown whether there exist causal dependencies between specific virus populations and whether the replication of individual virus populations is associated with mutual reduction or increase of other virus populations in the same microenvironment at a given time point or over time. Due to high genetic variability in *Anelloviridae*, it has been proposed that some *Torque teno viruses* may require complementary strains to gather all functionalities for successful infection and immune evasion [13]. This may generate possible liabilities between pathogenic and non-pathogenic viruses as well as the relation between different *Anellovirus* populations.

Especially in transplant recipients, potential virome cross-talk may have impact on the development of clinical complications, including microbial infections or transplant rejection, which limits transplant survival [11, 14]. In addition, as the total TTV load is increasingly used to measure the immunosuppressive net state after transplantation [15], knowledge about the distribution and potential interactions of different TTV genogroups within this patient setting needs to be further elucidated.

In the present study, we therefore investigated the quantitative relations between different virus populations in the virome of lung transplant recipients (LTRs), as well as their association with demographic or clinical parameters. We analyzed viral co-occurrence patterns and revealed robust clustering among specific virus groups in two different body compartments, i.e., lung and plasma. Importantly, we also studied cluster differences at specific time points after lung transplantation to characterize viral adaptation dynamics after solid organ transplantation.

## Methods

### Patient collective

In this exploratory study, samples from 49 patients were included, who all received a lung transplant at the Medical University of Vienna between 2012 and 2018 and from whom clinical specimens were available for virome analyses. In detail, we received 96 bronchoalveolar lavage (BALF) samples from 41 patients, as well as 45 plasma samples from 22 patients of which 14 patients provided both types of samples. Demographic and clinical data were recorded and all patients were followed up for at least 1 year. Patient demographics and samples are described in detail in SI Table 1A and B, respectively. All patients received induction therapy with 30 mg Alemtuzumab (Berlex), and maintenance therapy with tacrolimus (Prograf) and corticosteroids (Prednisolone). Additionally, all patients received antibiotic prophylaxis for two weeks, anti-fungal prophylaxis with aerosolized amphotericin B for 3 months, and HCMV-prophylaxis consisted of four doses of intravenous HCMV hyperimmunoglobuline during the first month and Val/ganciclovir administered for at least 3 months post-transplantation. The study was approved by the local Ethics Committee at the Medical University of Vienna (EK-number 1880/2016 and EK-number 1710/2014).

### Data generation

Data used for analysis comprise newly generated virome data derived from 68 BALF and 13 plasma samples from 34 lung transplant patients as well as previously generated virome data of 15 LTRs (Segura-Wang et al., 2018).

### Virus enrichment

Viral enrichment of BALF and plasma samples was done as described previously [4]. In short, 1 ml BALF aliquot was centrifuged for 5 min at 1200 g. Both, BALF and plasma volumes were adjusted to 1.2 ml with PBS and filtered through 0.45 μM filters (Sartorious). To remove cell-free DNA and RNA, the supernatant was treated with RNase A (QIAGEN) and TURBO DNase (Ambion) to final concentrations of 0.77 mg/ml and 20 Units/ml, respectively, incubated for 1 h at 37 °C, and deactivated by Protease (QIAGEN) treatment for 30 min at 37 °C.

### Nucleic acid extraction

Total nucleic acids were extracted using 1 ml of the filtered and nuclease treated material, with the automated NucliSENS EasyMAG system (BioMérieux).

### Metagenomic sequencing

For each extracted sample, RNA and DNA were amplified by random primer amplification in separate reactions and pooled before sequencing. RNA was reverse transcribed using a random octamer linked to an anchor sequence ATCGTCGTCGTAGGCTGCTCNNNNNNNN 15–18. Amplification reactions used 5 μl of extracted eluate and 5 μM random primers and were incubated for 5 min at 65 °C. Then 20 μl total volume reactions were set up as described in [16]. The resulting cDNA was denatured for 2 min at 94 °C, cooled 5 min at 10 °C, and used for second-strand synthesis. Similarly, the DNA from the extracted eluate was randomly amplified by using 5 μl of template. The resulting DNA derived from both, RNA and DNA templates, was subsequently amplified in separate reactions by PCR using 4 μl of input DNA from the previous step, 0.25 μM dNTP mix (Promega), 2 mM MgCl_2_, 1x AmpliTaq Gold Buffer II (Applied Biosystems), 0.05 U/μl AmpliTaq Gold Polymerase (Applied Biosystems), and 1 μM of an anchor specific primer for a total volume of 50 μl. The products were quantified by Qubit dsDNA BR Assay (Invitrogen) and diluted to 0.4 ng/μl. Equal amounts of the amplified and diluted cDNA and DNA were pooled at 0.4 ng/μl, and used as input for library preparation using the NexteraXT Library Preparation kit (Illumina). Samples were sequenced on an Illumina MiSeq platform, single-read or paired-read, 150bp and 250bp, respectively.

### Viral bioinformatics

Bioinformatic processing of the metagenomic sequencing data was performed with CLC Genomics Workbench 12.0 (QIAGEN Bioinformatics). Raw sequencing reads were trimmed including filtering for length ≥ 70 bp and removing low quality reads with an average Phred score below 30. Subsequently, the sequencing data were cleaned from non-viral reads by sequential mapping to a human reference genome (GRCh38), and to sets of bacterial and bovine reference genomes. To identify sequences of viral origin the remaining reads were aligned to a set of viral reference genomes by NCBI BLASTn [17] integrated into CLC Genomics Workbench. The used viral reference database was originally derived from the NCBI GenBank nuccore database [16, 18] which was cleaned from unverified isolates of TTV and thus consisted of 49,069 complete viral genomes including all 29 TTV genotypes officially recognized by ICTV [19] and verified TTV isolates according to NCBI GenBank [18]. BLAST search parameters included a minimum word size of 20, an *E* value ≤ 0.001 and filtering genetic regions with low entropy. For each sequenced read, the BLAST hit with the lowest *E* value was used to identify viral species. The identified sequences were further filtered to an *E* value ≤ 1E-15 and a species was reported provided that at least five reads were found in the filtered data set of a sample. TTV reads were further categorized to the respective *Alpha torque virus* genogroup according to their phylogenetic relationship, which was evaluated as described before [13].

### Data preparation

Sequencing counts were normalized against total metagenome counts to reduce compositional effects of viral species in subsequent analyses. The fold excess of total metagenome counts vs. total viral counts per sample amounted to 5367.2 on average. Subsequently, viral counts were normalized against the respective genome sizes according to [20] and technical replicates were averaged. To ensure robust abundance signals for downstream analyses, we denoised the data set and removed organisms with a presence of ≤ 10% in plasma and BALF samples, respectively and an average abundance per sample ≤ 0.01 according to [21]. The resulting read abundances were 4th root transformed to reduce data skewness for further analyses as in [22].

### Site-specific adaptation of lung and plasma virome after transplantation, exclusion dynamics

To clarify whether virome composition and abundance in the allograft and plasma converge over time, we evaluated the similarity of 56 matched sequencing samples from both body parts. In detail, first samples were categorized by time of collection after surgery using 5–12 weeks, 13–34 weeks, 35–95 weeks intervals. Then we calculated centroids from samples derived from both sides individually and compared its Bray-Curtis distance to all samples within and among sites. Centroids were calculated using R package usedist [23] and vegan [24]. Subsequently, all within site distances and between site distances were grouped and similarity of distributions was tested using Wilcoxon test statistics.

### Virome predictors and variable hierarchy

We investigated driving variables and their hierarchy in the data using independent and nested permutational multivariate analysis of variation (PERMANOVA). We tested sample variance with the categorical covariates patient and time after transplantation. Time was log-transformed to study proportional changes after transplantation and categorized into intervals (2–4 weeks, 5–12 weeks, 13–34 weeks, 35–95 weeks) that entered as factor into the statistical models. Sequencing data were 4th root transformed as in [22] and PERMANOVA was performed on sample Bray-Curtis distances using adonis2 from R package vegan [24]. Variable hierarchy was determined by iteratively nesting either covariate.

### Clusters of viral co-occurrence

We performed co-occurrence analyses to identify robust patterns among viral organisms using SparCC [25]. To correct for biases introduced by individual patients, we pursued a leave-one-out (LOO) strategy by iteratively removing samples derived from every patient resulting in 41/22 correlation matrices for BALF and plasma, respectively.

To identify the optimal number of clusters and to overcome technical limitations imposed by sparse and signed correlation matrices, we performed partitioning around medoids (PAM) of the full correlation matrices in parallel with graph clustering (blockmodels) of only strong correlations (rho>|0.2|, *p* < 0.05) on the core virome present in all graphs. The data were clustered with varying *k* = 3–10 and the resulting partitioning quality was assessed by complementary quality measures (Dunn Index, Dunn2 Index, Separation Index, Widest Gap, Pearson Gamma). Subsequently, the mean value for each measure was assessed across all 41/22 matrices and the most supported cluster number was identified by majority vote throughout these measures, for BALF and plasma independently. Partitioning and quality check was performed in R using the packages clusters, signet, fpc [26–28].

To assign core organisms to their final clusters, we harmonized the information on cluster association *α* obtained from pam partitions and blockmodels according to

$$\alpha \left(ij\right)={\sum}_0^N{c}_{ij}\left/ N\right.$$

with *c*_*ij*_ being instances where organisms *i* and *j* are classified to the same cluster within one partitioned set and *N* representing the total number of partitioned sets. PAM was performed on the resulting pairwise distance *d*(*i* *j*) = 1 − *α*(*i* *j*).

### Correlation networks

To correct for potential patient bias, we generated 41 BAL/22 plasma networks from the co-occurrence analyses described above using rho>|0.2| and *p* < 0.05 as cutoff for including edges into the graph. To integrate these into a single BALF and plasma graph, we first identified organism present in all respective graphs (core virome) and added edges, if present in > 50% of the graphs (majority vote). The average weight of the individual edges across all graphs is indicated in the final graph.

### Ordination of virome and association testing with genogroup

For the core virome, a single distance matrix for BALF and plasma was inferred by averaging over the LOO distance matrices derived from correlation analyses. Ordination was performed on averaged virome distances using PHATE [29] to ensure proper denoising and improved embedding of high dimensional correlation data. Variance testing of distances, genogroups, and interactions thereof was performed with PERMANOVA [24]. To test associations between viral genogroups and partitioned clusters, a *χ*^2^ test was performed on the contingency table of genotype frequency in clusters using R package psych [30]. The association among genogroups within clusters was assessed using Pearson correlation of the contingency table and Benjamini-Hochberg correction for multiple testing using corrplot [31].

### Linear mixed effect models for assessing drivers of virome structure

We analyzed the relationship of transplant recipient virome at the level of composition and richness with time and patient characteristics. We tested whether individual viral clusters can be significantly predicted by fitting a mixed effect intercept model (LMM) with following standard structure

$$\boldsymbol{y}=\boldsymbol{X}\boldsymbol{\alpha } +\boldsymbol{Z}\boldsymbol{\beta } +\boldsymbol{\varepsilon}$$

where *y* is a vector of the response variable, *X* is the vector of fixed effect predictor, *Z* is the matrix of random effects, and α, β, ε represent vectors of fitted regression coefficients.

The response variable was constructed as sum of 4th root transformed reads associated with the same cluster per sample or alternatively, as sum of different organism per cluster per sample normalized relative to maximum value achieved. For plasma-derived clusters, we used patient identity as fixed effect and log(time) after transplant as random effect. For BAL-derived clusters, both patient ID or log(time) were tested as fixed effects independently, with the respective other variable used as random effects. Subsequently, statistical significance of the dependence was determined by ANOVA testing on model F-statistic (type III analysis, Satterthwaite’s method) using packages lmerTest [32] and core package stats. For visualization of effect sizes, model fits, and dynamics over time, we used effects [33], ggplot2 and ggpubr [34], respectively.

For detecting driver organism in time and for TX indication, denoised and clr transformed read data were used as response variable and log(time)/indication categories were used as fixed effects; patient and batch ID were included as random effects.

### Spatial exclusion patterns in the lung

To quantify exclusion of particular viral organism in the same BALF sample, we compared the 4th root transformed abundances of viruses in time-associated lung clusters and defined the degree of segregation between the community clusters. The viral abundances were summed by cluster and their ratios stratified into 5 exclusion categories, these are presence of organisms of only one cluster (100% exclusion), presence of organisms of both clusters: abundance ratio ≤ 1:4 (75%exclusion), ≤ 1:2 (50%exclusion), ≤ 3:4 (25% exclusion), and comparable abundance levels (no exclusion). Sample numbers according to exclusion categories are presented (*n* = 56).

### Association of patient groups and clinical meta-information

For LMM models, where a significant association of viral clusters with patient identity was detected, patients were split into 2 subgroups based on their estimated regression parameters α (fixed effects). In detail, patient groups were formed from regression parameters with different sign (+/−). Subsequently, we tested association of these patient groups with clinical meta-information, i.e., recipient TX indication, donor CMV status, acute rejection episodes, induction therapy, immuno-suppressive treatment categories, and recipient sex. Contingency tables were inferred, categories represented with a single instance were removed, and *χ*^2^ test was performed to assess association and statistical significance.

## Results

### Virome structure in lung and blood develops independently after lung transplantation

**Fig. 1:**
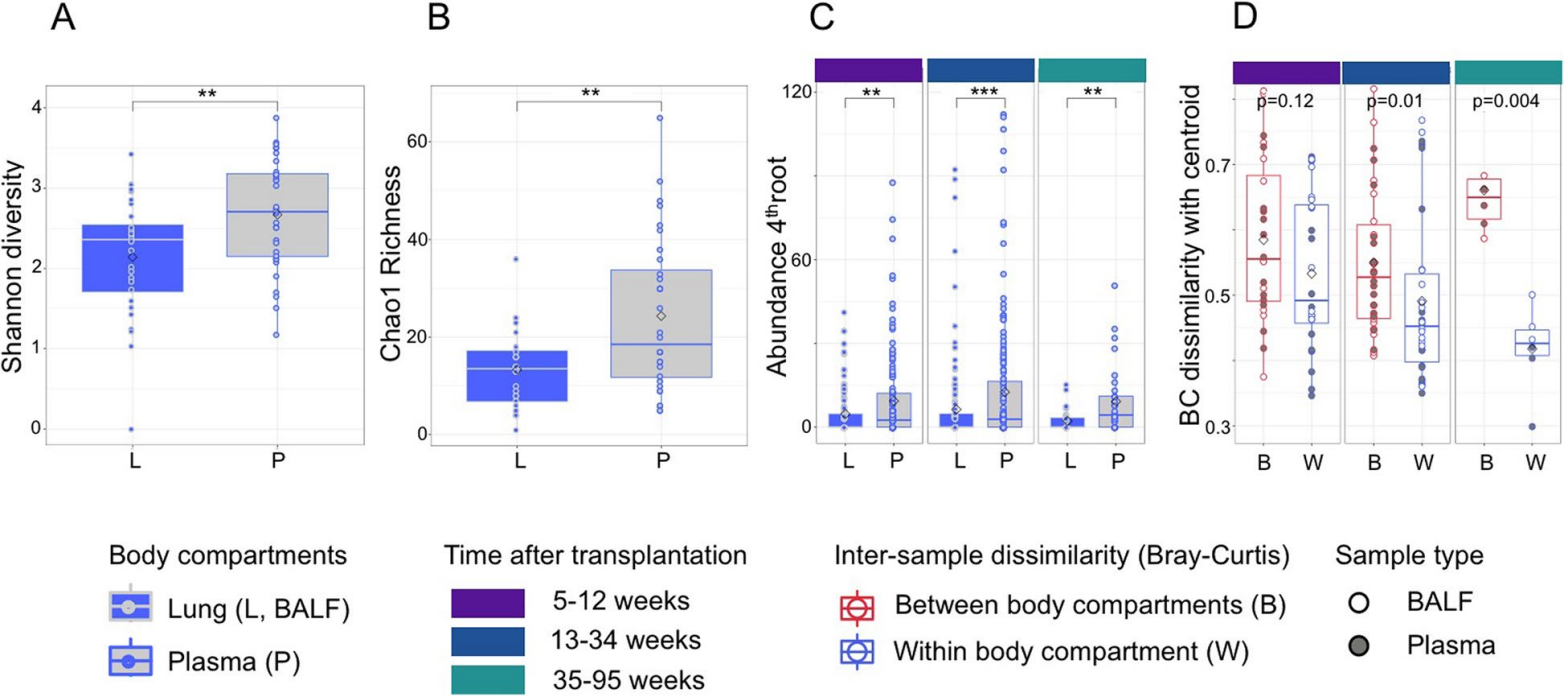
Body-site specific virus ecology after lung transplantation (sample numbers: n_L_ = 28, n_P_ = 28). **A** Shannon diversities of lung and blood derived viruses in LTRs, 56 BALF and plasma samples collected from 14 patients on the same day, species level annotation. Distribution differences between body-sites **p < 0.01, Wilcoxon-Mann-Whitney test. **B** Chao1 richness index of lung and blood derived virome (matched samples as above). Distribution difference **p < 0.01, Wilcoxon-Mann-Whitney test. **C** Change of viral sequence abundance in matched BALF and plasma samples facetted by time after lung transplantation (5–12 weeks, 13–34 weeks, 35–95 weeks). Sequencing counts were normalized against total metagenome counts and individual viral genome sizes, the resulting abundance indices were 4th root transformed [Aziz et al Front Microbiol 2015]. **p < 0.01, ***p < 0.001. **D** Inter-sample dissimilarity within (W) and between (B) body sites facetted by time after transplantation (matched samples as above). Shown are Bray-Curtis dissimilarities among samples and body-site specific sample centroids. Filled circles indicate plasma, white circles BALF samples. *P* values are depicted, Wilcoxon-Mann-Whitney test

To better understand how the recipient virome rearranges after lung transplantation and to determine relevant drivers in this process, we analyzed the variation of viral composition and abundance in matched samples; these were 28 BALF and 28 plasma samples collected at the same day from 14 patients based on metagenomic sequencing. The virome was annotated to species level and as it was strongly dominated by eukaryotic viruses, only these were included in the analyses. Overall, *Anelloviridae* dominated the virome composition (*Anelloviridae*:*non*-*Anelloviridae* = 16:1) with *Alpha torque viruses* (TTV) belonging to genogroup 3 being most abundant in 70% of the samples. We observed that blood and lung-derived viromes differed significantly (Fig. 1). Shannon diversity and Chao1 richness both indicated a significantly higher taxonomic diversity in plasma samples compared to BALF (Fig. 1A, B). Similarly, the viral abundance at different, matched time intervals after TX (5–12 weeks, 13–34 weeks, and 35–95 weeks) was significantly higher in plasma than in BALF at all time points tested (Fig. 1C).

We further asked whether the site-specific differences adjusted over time, i.e., whether the viromes of blood and alveolar compartment became more similar with time after transplantation. To this end, we determined the centroid composition of all plasma and BALF samples within three defined time periods independently, and tested the temporal changes in Bray-Curtis dissimilarity of samples to centroid, both within and between body sites (Fig. 1D). The results showed that differences between plasma and airway virome persisted and even increased over the first year after transplantation, whereas samples from the same body site became more alike over time, suggesting the development of a sampling site specific viral community.

To test whether these site-specific virus variations can be explained by temporal changes after transplantation (time-dependency), by patient identity, or by a combination of both, we performed permutational multivariate analysis of variance (PERMANOVA). We included all 141 study samples to increase the statistical power, i.e., 45 plasma, 96 BALF samples (detailed data description in SI Table 1). Again, lung-derived and blood-derived viromes yielded different results (SI Table 2A). While both variables yielded statistically significant results if tested independently, we identified hierarchical effects by nesting the variables iteratively. In this limited data, patient identity accounted significantly for variance at both sites (plasma: *R*^2^ = 0.67, *p* < 0.001; lung: *R*^2^ = 0.63, *p* < 0.001), while time was significantly associated with lung (*R*^2^ = 0.09, *p* < 0.002) but not plasma (*R*^2^ = 0.14, *p* = 0.2) virome variance. These trends persisted also if the number of lung samples was reduced to the number of plasma samples (*n* = 45, 1000 random subsamples, SI Table 2B).

Together, our analyses suggested that the lung virome underwent detectable changes with time after transplantation. For both body sites, patient-specific factors robustly impacted sample variance.

### Co-occurrence analysis reveals viral clusters that delimit genetic groups

**Fig. 2:**
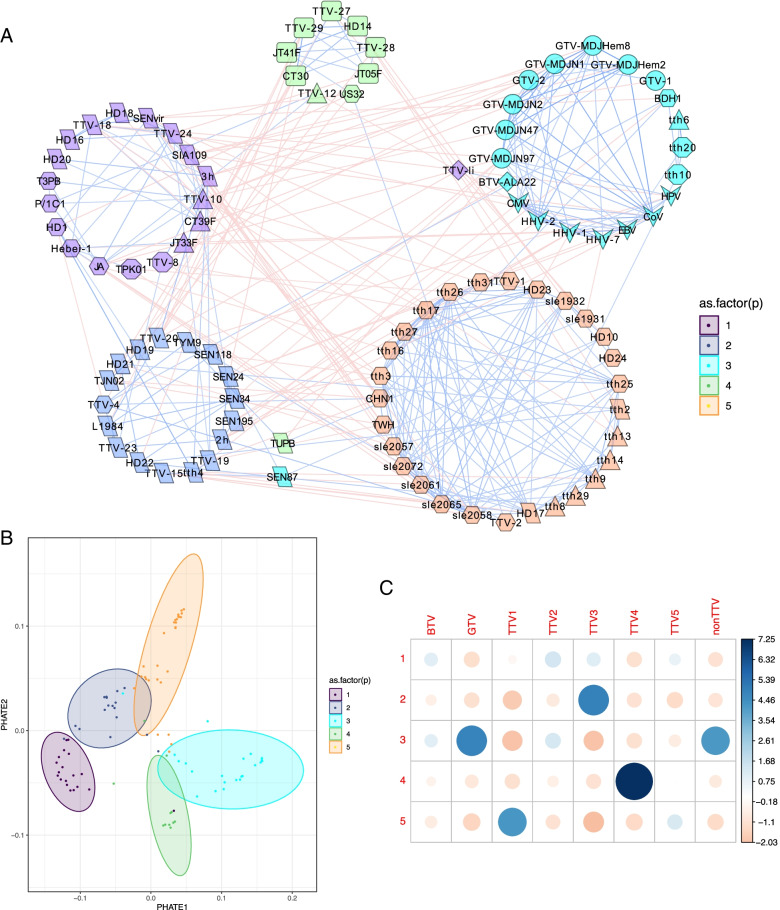
*Anelloviruses* with same genogroup tend to form community clusters in the lung (Sample number: n_L_ = 96). **A** Co-occurrence network of viruses in the lung. Nodes represent core viral species shaped according to TTV genogroups (TTV1-5), *Beta*- (BTV) and *Gamma torque virus* (GTV) or *non*-*Anelloviruses* (nonAnello), edges depict correlation (red) and exclusion (blue). Networks were calculated iteratively removing every patient (leave-one-out strategy) to correct potential patient bias. The fused graph depicts viruses present in all subnetworks (core virome), edges present in > 50% of subnetworks, edge weight as average over the respective values in the subnetworks. **B** 2-dimensional PHATE ordination of distances between core viral strains in the lung virome. Depicted clusters were inferred from partitioning around medoids of correlation matrix combined with block models of graph adjacency matrices. *p* < 0.001, PERMANOVA. **C** Association of *Anelloviruses*, genogroups and pathogenic viruses with viral clusters in the lung. BTV *Beta torque virus*, GTV *Gamma torque virus*, TTV1-5 *Alpha torque virus* genogroups, nonAnello *non*-*Anelloviridae*, Pearson *χ*^2^ test, association strength color-coded, circle size depicts normalized strength, *χ*^2^ = 185, *p* < 0.0001

**Fig. 3:**
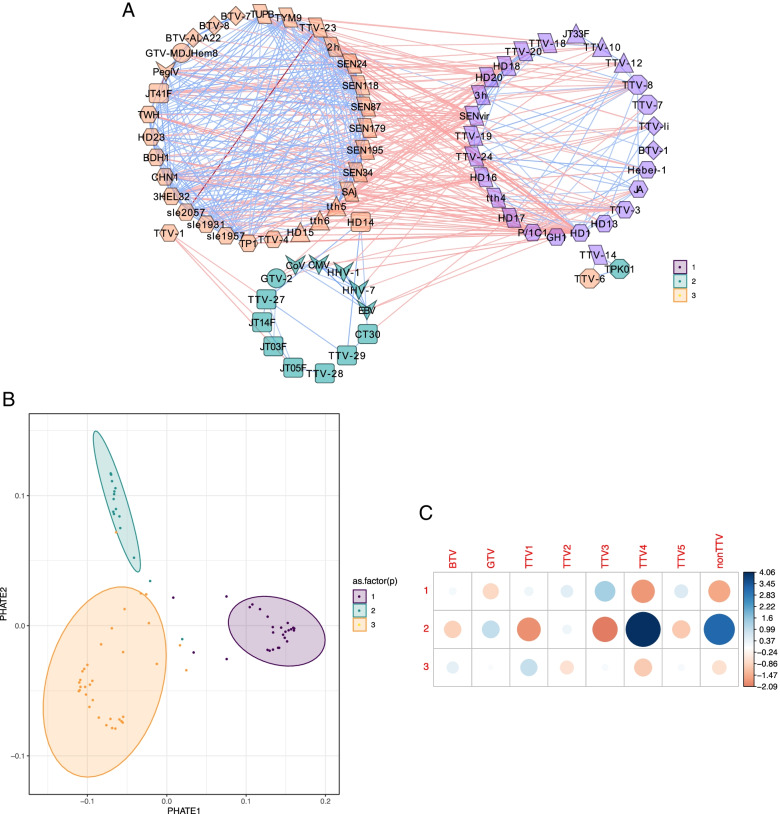
Virus community clusters in plasma (sample number: n_P_ = 45). **A** Co-occurrence network of viral abundances in patient plasma. Nodes represent core viral species shaped according to TTV genogroups (TTV1-5), *Beta-* (BTV) and *Gamma torque virus* (GTV) or *non-Anelloviruses* (nonAnello), edges depict correlation (red), and exclusion (blue). Networks were calculated iteratively removing every patient (leave-one-out strategy) to correct potential patient bias. The fused graph depicts viruses present in all subnetworks (core virome), edges present in > 50% of subnetworks, edge weight as average over the respective values in the subnetworks. **B** 2-dimensional PHATE ordination of distances between core viral strains in the plasma virome. Depicted clusters were inferred from partitioning around medoids of correlation matrix combined with block models of graph adjacency matrices. *p* < 0.001, PERMANOVA. **C** Association of *Anellovirus* genogroups and pathogenic viruses with viral clusters in blood. BTV *Beta torque virus*, GTV *Gamma torque virus*, TTV1-4 *Alpha torque virus* genogroups, nonTTV pathogenic viruses. Pearson *χ*^2^ test, association strength color-coded, circle size depicts normalized strength, *χ*^2^ = 50, *p* < 0.0001

Next, we analyzed the co-occurrence patterns of viral species after transplantation. Due to the dynamical and compositional differences among body sites, we conducted an independent analysis on 45 plasma and 96 BALF samples, respectively, including all time points of collection. To correct for potential biases due to individual patients, we iteratively removed individual patients from the analysis (leave-one-out stratification). Interestingly, we observed strong exclusive viral communities characterized by positive association within and negative associations between groups of virus species in BALF (Fig. 2A) and plasma (Fig. 3A). To better discriminate the correlation patterns, we performed a cluster analysis that accommodated the particular properties of co-occurrence matrices; these are positive and negative (signed) association values, and sparsity due to weak correlations. We performed cluster analysis on blockmodels of sparse and signed adjacency matrices and combined the outcome with results from standard partitioning around medoids (PAM) clustering on quantitative distance matrices. We found five strong community clusters in the lung and three in plasma (PERMANOVA *p* < 0.001 in both sites) (Fig. 2B, Fig. 3B), whose composition was strongly defined by different *Anelloviruses*. We performed *χ*^2^ testing to examine the association of individual virus species with clusters (Fig. 2C). In the lung, TTV species belonging to genogroup 1 (TTV1), genogroup 3 (TTV3), and genogroup 4 (TTV4) dominated their respective clusters and pathogenic viruses like Coronavirus, Cytomegalovirus, and other human Herpesviruses co-occurred with *Gamma torque viruses* (GTV) in a separate cluster (*χ*^2^ = 185,2, *p* < 0.0001).

In the blood compartment, the association of pathogenic viruses with GTV persisted, but was extended by co-occurrence of TTV4 in the same cluster (Fig. 3C). The other two clusters determined in plasma presented mixed communities comprising TTV1, 3, 5, and *Beta torque viruses*, respectively (*χ*^2^ = 49,7, *p* < 0.0001). We confirmed the associations among *Anellovirus* subpopulations and *non*-*Anelloviruses* in parallel by Pearson correlation for BALF samples (SI Fig. 1A) and plasma samples (SI Fig. 1B). These results highlight a distinct and significant association between specific *Anellovirus* subpopulations and between these and pathogenic viruses in the different compartments.

### Viruses display specific temporal dynamics in the lung

**Fig. 4:**
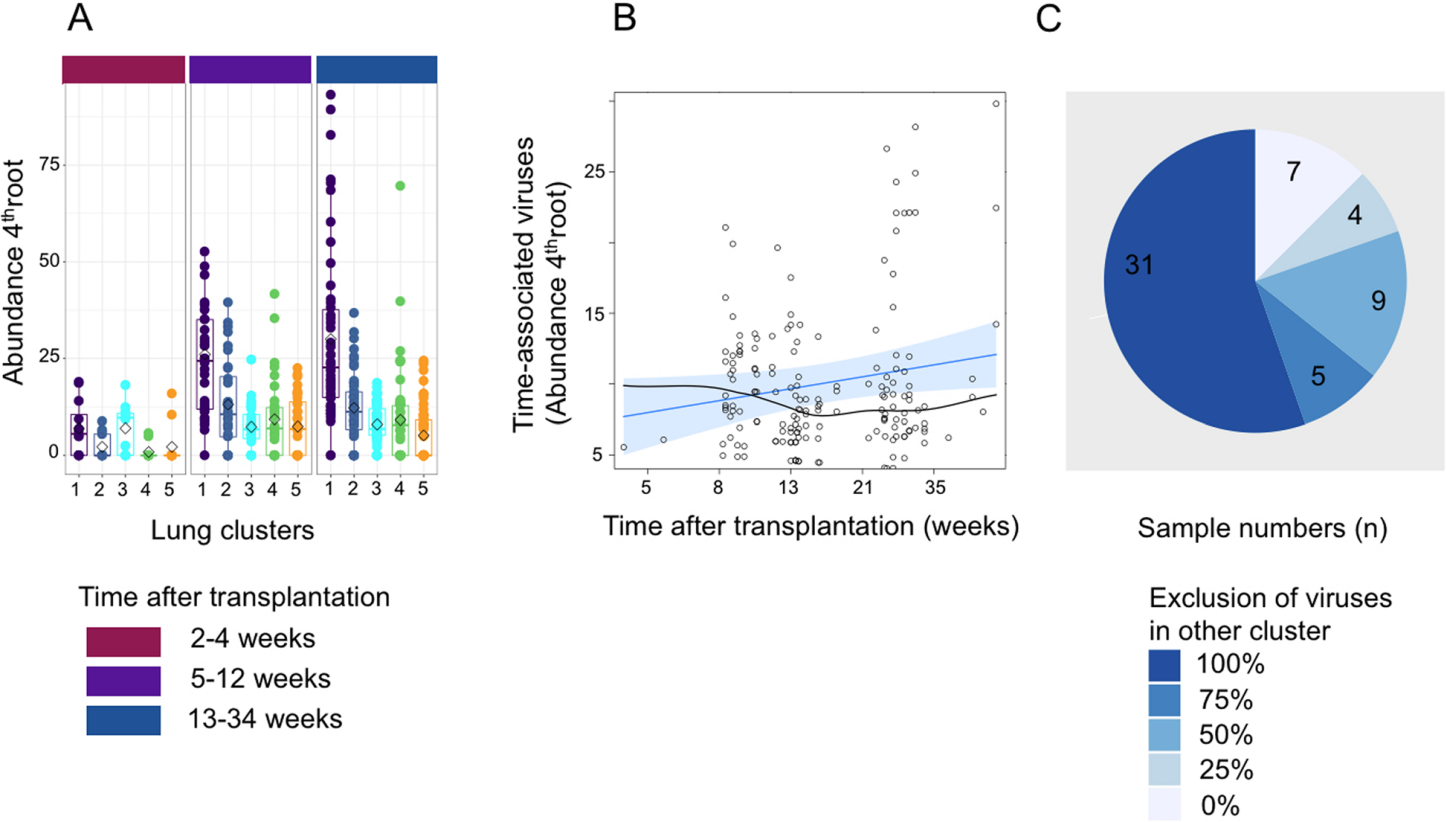
Temporal and spatial behavior of the lung virome. **A** Abundance change of viral lung clusters over time, core viral species abundances summed by cluster and 4th root transformed. Sample number: n_L_ = 96. **B** Linear mixed model of time-associated viruses across lung clusters 1, 2, 4. Data points (circles), linear regression (blue line), and residuals (black line) are depicted. Weeks after surgery are depicted in log scale. Sample number: n_L_ = 90. **C** Cluster-based exclusion of virus in same BALF sample. 4th root transformed abundances of time-associated organism in same cluster (2 or 4) were summed and ratio of their abundances in the same sample was used to assign exclusion categories 0:1 = 100%, < 1:4 = 75%, < 1:2 = 50%, < 3:4 = 25%, and no exclusion. Increasing sample counts are depicted. Sample number: n_L_ = 56

To dissect in detail the viral adaptation in the lung compartment over time after transplantation, we analyzed the changes in abundance of the viral sequences in the five virus clusters found in the lung over the first 8 months post transplantation. Therefore, we built linear mixed effect models (LMM) on the 96 BALF samples, to identify which of the five clusters significantly associated with log-transformed continuous time after transplantation, while introducing patient identity as random effects to the model (Fig. 4A, SI Fig. 2A–C). We discovered that only clusters 1, 2, and 4 displayed a significant increase in viral abundance over time (cluster nomenclature as depicted in Fig. 2). These clusters were rich in species belonging especially to TTV genogroup 3 or 4 (LMM, *p* < 0.05). On the level of single virus strains, SEN34, HD21, HD22, 19, 27, 29, L1984, and JT05F displayed a significant time-associated increase (LMM, *p* < 0.05, Fig. 4B). The results highlight that virome adaptation did not proceed uniformly within the *Anelloviruses* and perturbation of the microenvironment in the lung compartment after transplantation seemed to benefit replication of certain virus strains over others.

We further asked whether these time-associated TTV strains increased simultaneously or whether increase of one strain prohibited the emergence of others. We found that only those strains associated with the same cluster increased simultaneously, while strains from different clusters were not observed together at high levels. In 80% (45 out of 56) BALF samples, the dominant viruses where more than twice as abundant in the sample as viruses confined to the inferior cluster.

### Recipients’ underlying disease imprints long-term plasma virome structure

**Fig. 5:**
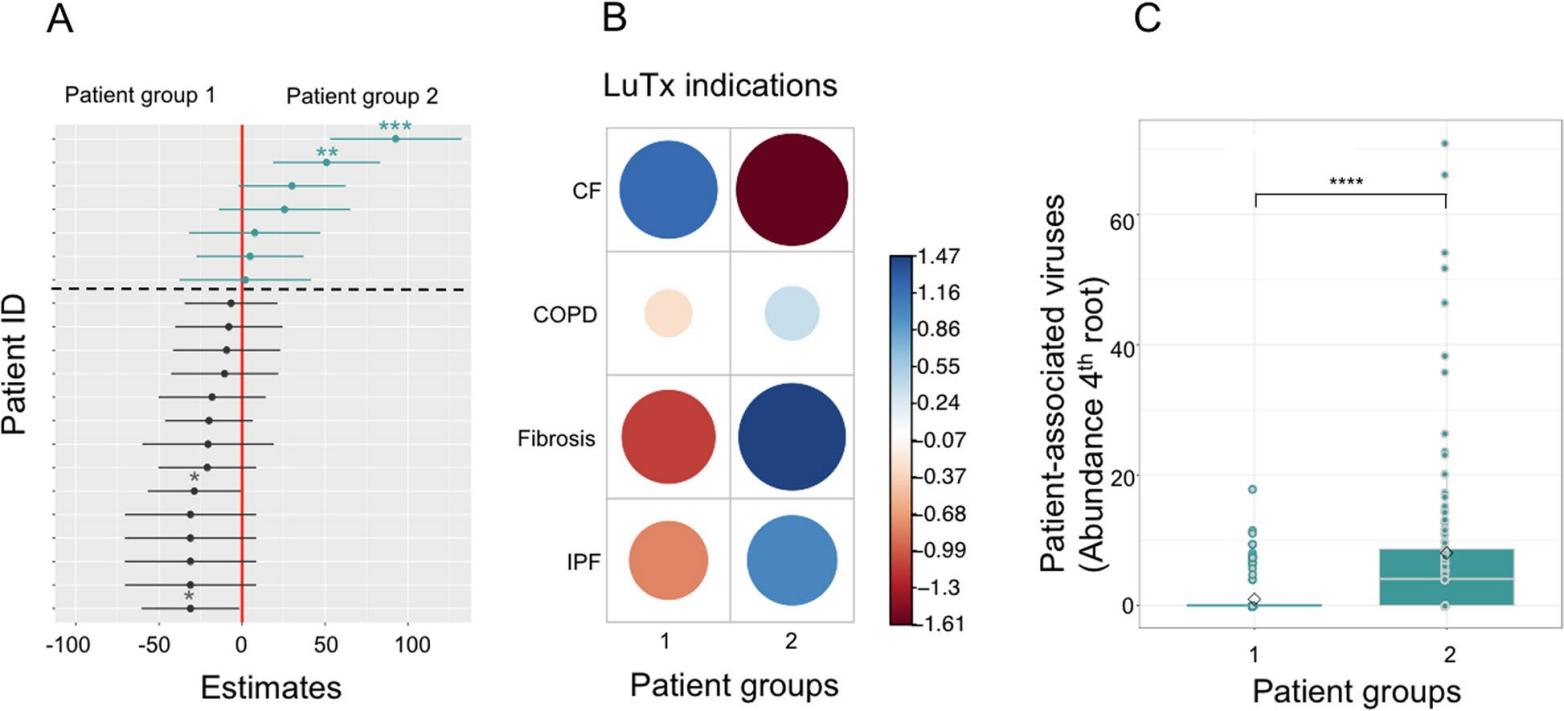
Patient-associated drivers of plasma virome structure. **A** Estimated fixed effects of linear mixed effect regression on plasma cluster 3 (LMM, *p* < 0.05). Patient ID was used as explanatory variable, cluster abundance as response, and time after transplantation entered as random factor. The fixed effect estimates were colored according to their sign (turquoise, positive value, gray negative values), significance levels were indicated. The model fit splits patients into two patient groups (broken line). Sample number: n_P_ = 45. **B** Association of underlying diseases with LMM patient groups. Cystic fibrosis (CF), chronic obstructive pulmonary disease (COPD), pulmonary fibrosis (Fibrosis), idiopathic pulmonary fibrosis (IPF). Association strength was color coded, size depicts normalized strengths, *χ*^2^ =9.5, p=0.02. Patient number: n_S_ = 19. **C** Differential abundance of patient-associated viruses in LMM patient groups. Abundances were 4th root transformed. Wilcoxon, *p* < 0.0001. Sample number: n_P_ =30

Based on the initial finding that virome variation in plasma was affected not by time but by patient identity, we sought to identify clinical parameters that may explain differences in recipient virome variations in an exploratory manner. To this end, we modeled viral cluster abundance in 45 plasma samples with linear mixed effect models using patient ID as explanatory variable and time after transplantation entered as random effects. Only plasma cluster 3 showed a significant linear relationship with the underlying patient identity (LMM, *p* < 0.05). Analysis of the model parameter estimates revealed that these split the patient collective that have viral species belonging to cluster 3 (dominated by TTV1 and BTV) into two groups with high or low abundances, respectively (Fig. 5A, C). In a next step, we asked, which patient-related factors might be associated with the grouping. We tested group association with clinical categories including indication for transplantation, survival, and prevalence of chronic lung allograft dysfunction (CLAD) performing *χ*^2^ tests. In our limited data set, we found that solely the underlying disease significantly specified the patient groups (*p* = 0.024, *p* = 0.004, respectively, Fig. 5B). As such, patients originally diagnosed with chronic obstructive pulmonary disease (COPD), fibrosis, or idiopathic pulmonary fibrosis (IPF) presented a different plasma virome than patients with CF. As lung recipients with CF tended to be younger than recipients with different diagnosis, we assessed whether patient age could explain patient grouping in a similar manner (SI Fig. 3). The resulting association between age and patient grouping shows a positive trend *χ*^2^ = 5.949, but misses statistical significance (*p* = 0.051). While an explorative association analysis like this one cannot reinforce causal conclusions nor enable to distinguish drivers in a set of covariates like CF and age, our results underline the importance of host factors for the long-term dynamics of the human virome.

## Discussion

The human virome is intimately related with the health status of its host. Viral populations colonize the neonate shortly after birth and develop a body site specific signature later on [35]. While the adult virome is relatively stable in individual body compartments of a person over time [36], the transplantation of an organ from a donor to a recipient organism leads to the introduction of an unrelated viral population to a host. It is unclear how such a perturbation affects viral population dynamics in the allograft and in other body compartments of the recipient.

The present study on a cohort of LTRs analyzing eukaryotic viruses in 141 plasma and BALF samples revealed compartment-specific viromes that display separate dynamics.

The analysis of inter-sample distances over time demonstrated that these differences between recipient blood and transplanted lung-derived virome not only persisted but even increased over the first year after transplantation. This modeling approach is in line with previous clinical observations in LTRs that detected the transfer of *Anelloviruses* from donor to recipient within the lung [37], but showed no clear imprint of the donor *Anellovirus* population on the recipient blood virome after kidney transplantation [38]. Thus, the data suggest that the *Anellovirus* dominated viromes of different hosts do not significantly interchange after solid organ transplantation.

Under healthy conditions, host antiviral immune responses exert control over persisting viruses by suppressing viral replication. Immunosuppressive therapy after transplantation releases this balance resulting in increased virus loads in LTRs, with *Anelloviridae* comprising a dominant fraction thereof [5]. A main question of the present study was to clarify whether epidemiologic or ecologic dependencies existed among viruses and viral strains that could become visible during such viral re-activations after transplantation. An important finding was the significant co-exclusion observed in the blood compartment between pathogenic viruses on one and *Alpha*- and *Beta torque virus* populations on the other hand. Earlier work showed similar epidemiological features of *Alpha*- and *Beta torque virus* strains in the context of acute respiratory diseases [39]. One possible explanation for the observed clustering and the co-exclusion with pathogenic viruses may be that we observe the result of viral adaptation to its microenvironment [40, 41]. Replication of pathogenic viruses such as *Herpesviruses* or *Hepatitis C Virus* leads to immune responses which elicit substantial cytokine expression [42]. These high cytokine levels in the environment may indirectly cause also a rapid limitation of *Anellovirus* replication affecting the majority of *Alpha*- and *Beta torque viruses*. In fact, it was described that therapeutic administration of alpha interferon due to specific diseases caused a secondary decrease of *Alpha*- and *Beta torque virus* levels [43, 44]. *Anelloviruses* themselves do most likely not induce high cytokine levels in the human host.

Interestingly, the pathogenic viruses in the studied plasma samples formed a strong synergistic cluster with GTVs and a specific subgroup of TTV, namely genogroup 4. Therefore, the question arose, whether TTV genogroup 4 is different in some way to other TTV genogroups. Borkosky and colleagues [45] discovered a functional relation between *Herpes* and *Anelloviridae* and showed that co-infection with *EBV* activated the replication of genogroup 4 isolate TTV-HD14 in vitro. Moreover, there are limited previous data, suggesting that the TTV4 genogroup may eventually be more pathogenic than other *Anelloviruses* [46]. The group of Maggi found increased TTV4 loads in children with acute respiratory diseases developing complications such as severe bronchopneumonia [39]. Whether these effects are evidence for a direct pathogenic effect is unclear, but fit the current findings that point toward a special status of TTV4 compared to other *Alpha torque viruses*. While *Anelloviruses* are in general seen as commensal, non-pathogenic viruses, the high proportion of *Anellovirus* co-infections may hide the fact that on overall one or the other member of this group may have some pathogenic potential or benefit from pathogenic virus infections. It has been shown that TTV and several *Herpes viruses* target similar blood cells in the human host, specifically monocytes and lymphocytes [2, 47, 48]. Thus, there may exist overlapping ecological niches of TTV and pathogenic viruses, which are possibly different between individual *Anellovirus* populations. This could also explain the clustering between pathogenic viruses and GTVs. While GTVs were found frequently in nasopharyngeal secretions of children with acute respiratory disease and also in the context of lung transplantation [49], it is supposed that they are innocent bystanders more than pathogenic agents [50].

In the lung, the level of viral replication was significantly associated with time after transplantation. Five viral clusters were identified with a more pronounced co-occurrence pattern among *Anellovirus* genogroups than in plasma. At the level of the population, it has been proposed that only few TTV strains possess all functional components to persist in the host, therefore co-infection is frequently observed to gather the required components [13, 46]. The higher number and definition of clusters in the BALF samples compared to plasma may be caused by a temporal instability or re-activation of the donor-derived lung virome. While the precise mechanisms remain obscure, the robust clustering of specific virus groups suggests that viruses within one cluster display similar activity in response to the local environment, which is distinct from viruses in antagonistic clusters. Moreover, the perturbation of the microenvironment after transplantation seems to benefit replication of certain virus strains, which at the same time preclude the increase of others in a predictable way.

Regarding the more pronounced time dynamics in the airways, the modeling presented in this retrospective study accurately reflects the characteristics of the studied data. However, the direct comparison between body compartments at early time points and potential temporal dynamics in plasma are limited due to lack of early, matched plasma samples in the collection and resulting sample size differences. Therefore, it cannot be excluded that also the plasma virome exhibits time dynamics after lung transplantation. We recommend to investigate the time behavior of the post-transplant plasma virome dynamics in an increased sample collection.

An important clinical question is whether the abundance of specific TTV strains or clusters can be linked to specific clinical complications after transplantation. We tested potential correlations with patient survival, the prevalence of CLAD development, and CMV state of donor and recipient. From these analyses, no significant drivers could be identified, but confirmation by a much larger patient collective would be required. However, we observed significant association between specific clusters in the blood compartment of LTRs and clinical background. Interestingly, patients with CF developed a significantly higher level of the TTV3 cluster than patients with COPD or fibrosis. Of note, the CF patient group tended to be younger than other LTRs, which could imply a potential role of age-dependent virome patterns in the lung [51]. Data correlation could not distinguish between these co-varying predictors; therefore, further validation in an extended patient cohort, with specific investigation on the underlying reasons is needed.

## Conclusions

Our study on a limited data set provides further evidence for mutual dependencies among different *Anelloviridae* and other opportunistic viruses that result in specific virome signatures that can be linked to host parameters, body site and time after lung transplantation.

## Supplementary Information

**Additional file 1: SI Figure 1**. Association of *Anelloviruses* and non-Anelloviruses in lung and blood compartments. **SI Figure 2**. Temporal changes of community cluster abundances show significant time-association in the lung. **SI Figure 3**. Association of age with patient grouping. **SI Table 1**. Description of patient demographics (A) and samples (B) used in this study. **SI Table 2**. Data structure and variable hierarchy.

## Data Availability

Metagenomic sequencing data as well as meta information were deposited at NCBI’s Sequence Read Archive under the BioProject accession number PRJNA764588 with individual sample accessions SAMN21511896-SAMN21512078. R code supporting the findings of this study was made available as supplementary material.

## Abbreviations

LTR: Lung transplant recipients
COPD: Chronic obstructive pulmonary disease
IPF: Idiopathic pulmonary fibrosis
CLAD: Chronic lung allograft dysfunction
TTV: *Alpha torque teno virus*
1-5 TTV1-5: *Alpha torque teno virus* genogoup
BTV: *Beta torque teno virus*
BTV: *Gamma torque teno virus*
BALF: Bronchoalveolar lavage fluid
n_P_: Number of plasma samples
n_L_: Number of BALF (lung) samples
n_S_: Number of subjects
