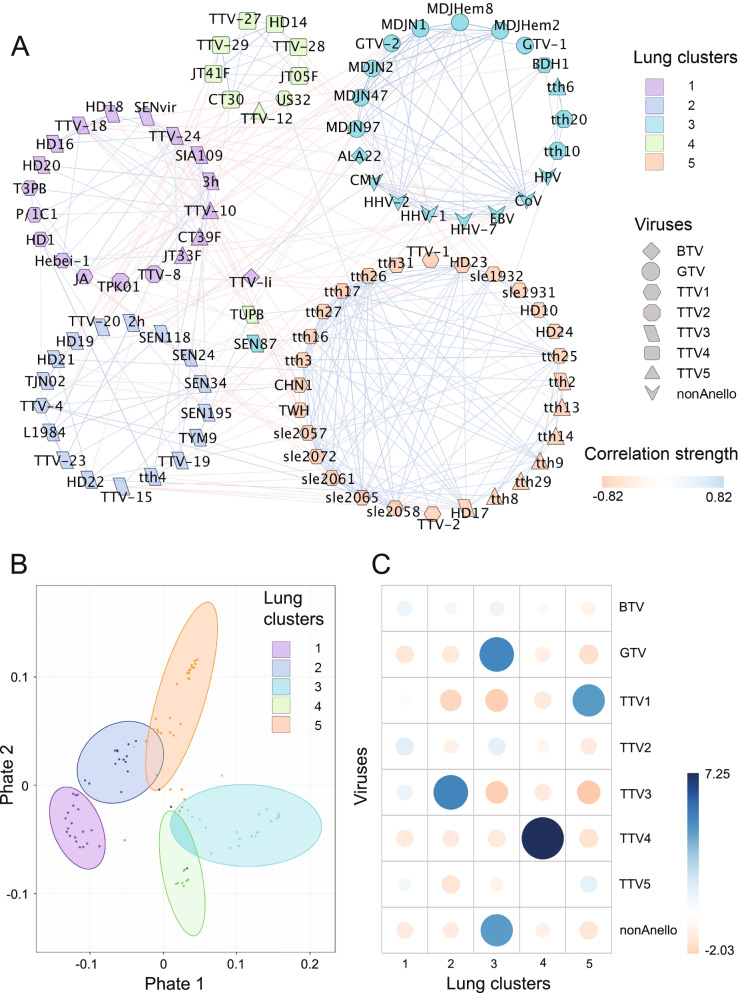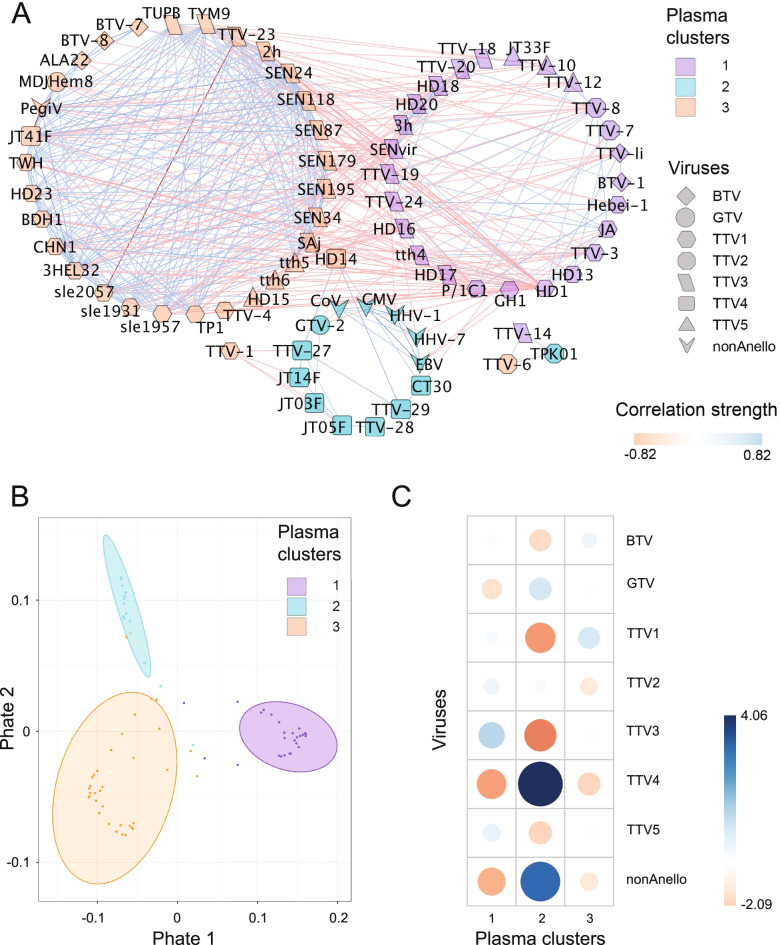# Correction: Metagenomic sequencing reveals time, host, and body compartment-specific viral dynamics after lung transplantation

**DOI:** 10.1186/s40168-022-01285-0

**Published:** 2022-05-30

**Authors:** Stefanie Widder, Irene Görzer, Benjamin Friedel, Nina Rahimi, Stefan Schwarz, Peter Jaksch, Sylvia Knapp, Elisabeth Puchhammer-Stöckl

**Affiliations:** 1 Research Laboratory of Infection Biology, Department of Medicine I, Medical University of Vienna, Vienna, Austria; 2 Konrad Lorenz Institute for Evolution and Cognition Research, Klosterneuburg, Austria; 3 Center of Virology, Medical University Vienna, Vienna, Austria; 4 Department for Internal Medicine, Diabetology, Endocrinology, Diakonissenkrankenhaus, ViDia Kliniken, Karlsruhe, Germany; 5 Division of Thoracic Surgery, Department of Surgery, Medical University of Vienna, Vienna, Austria

**Correction: Microbiome 10, 66 (2022)**

**https://doi.org/10.1186/s40168-022-01244-9**

Following the publication of the original article [1], the author reported that Figs. 2 and 3 were wrongly compiled. The correct figures are included here and the original article has been updated.