# Supplementary material for: Metagenomic sequencing reveals time, host, and body compartment-specific viral dynamics after lung transplantation
Source: Microbiome. 2022 Apr 23;10:66. doi: 10.1186/s40168-022-01244-9 (PMC9033415; doi:10.1186/s40168-022-01244-9)
Supplement: Supplementary file 2 — Additional file 1: SI Figure 1. Association of Anelloviruses and non-Anelloviruses in lung and blood compartments. SI Figure 2. Temporal changes of community cluster abundances show significant time-association in the lung. SI Figure 3. Association of age with patient grouping. SI Table 1. Description of patient demographics (A) and samples (B) used in this study. SI Table 2. Data structure and variable hierarchy. [file 40168_2022_1244_MOESM2_ESM.docx]

**(SI Figure 1)**
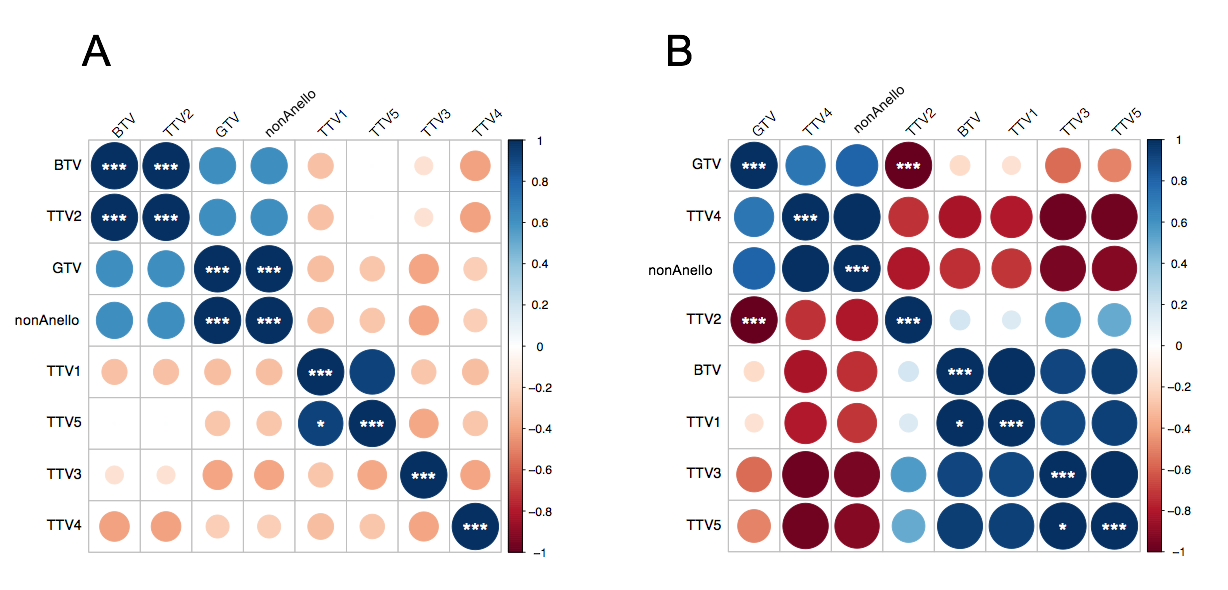


**SI Figure 1 Association of *Anelloviruses* and non-Anelloviruses in lung and blood compartments.**

**(A)** Correlation of *Anelloviruses* and pathogens in lung clusters. TTV genogroups (TTV1-5), *Beta-* (BTV) and *Gamma torque virus* (GTV) and nonAnelloviruses (nonAnello) are depicted. Pearson correlation and FDR correction by Benjamini-Hochberg method was performed on contingency table of virus groups on clusters, correlation strength (color, size) and significance levels (stars) are depicted. Sample number: n_L_=96

**(B)** Correlation of *Anelloviruses* and pathogens in plasma clusters. Pearson correlation and FDR correction by Benjamini-Hochberg method was performed on contingency table of virus groups on clusters, correlation strength (color, size) and significance levels (stars) are depicted. Sample number: n_P_=45

**(SI Figure 2)**

**
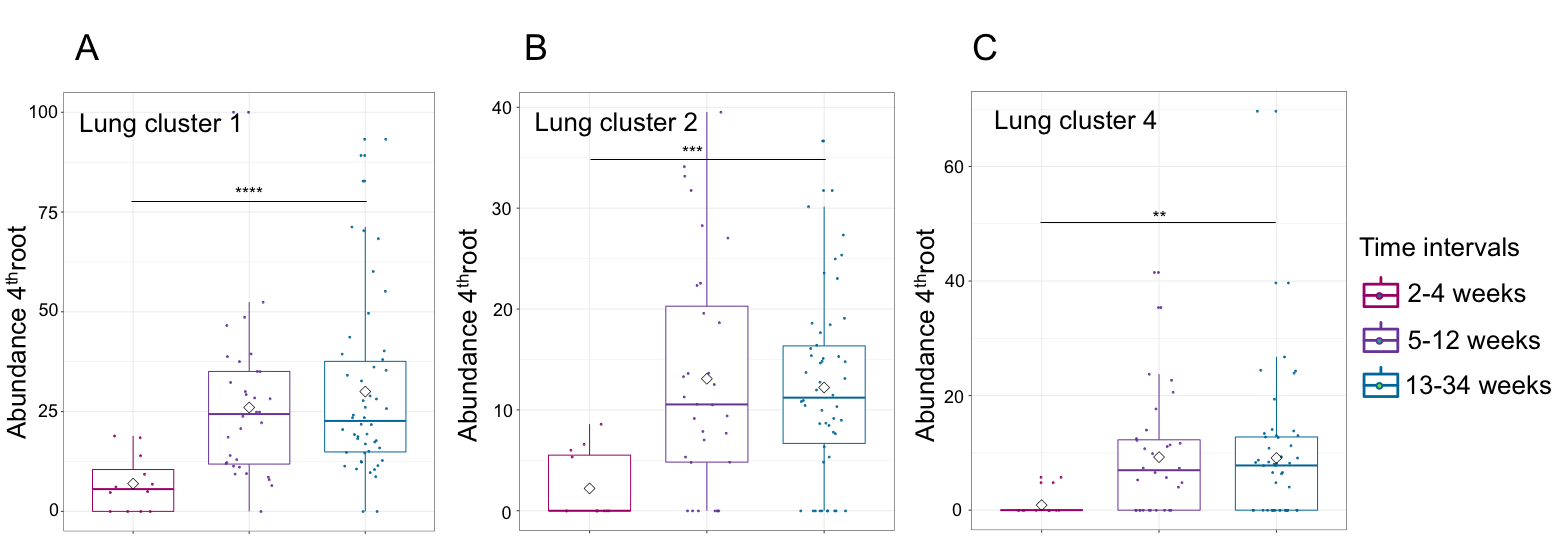
**

**SI Figure 2 Temporal changes of community cluster abundances show significant time-association in the lung.**

(A-C) Individual virus abundances were summed by cluster-association and 4^th^ root transformed. Time-association was assessed by LMM, p<0.05 for depicted clusters. Boxplots show quantiles and mean (diamond) of cluster abundances for three time intervals (2-4 weeks, 5-12 weeks, 13-34 weeks). Sample number: n_L_=90

**(SI Figure 3)**

**
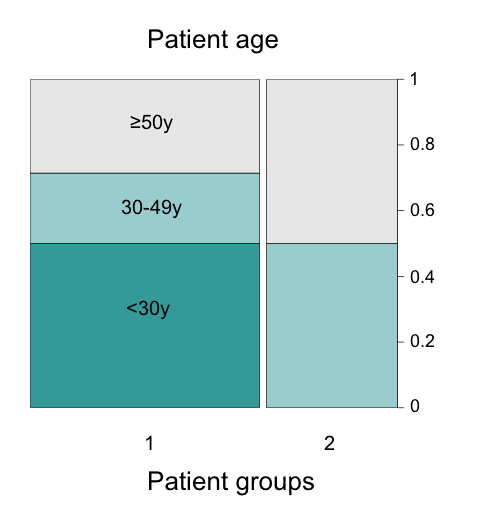
**

**SI Figure 3 Association of age with patient grouping.** Distribution of patient age at time of transplantation was stratified into 3 categories (18-30, 30-49, 50-63 years with n_1_=7, n_2_=7, n_3_=8, respectively) and association of age classes with patient grouping (Figure 5) was assessed by $\chi^{2}$ testing. Resulting variable association strength was $\chi^{2}$=5.949, p = 0.051. Subject number: n_S_=22

**(SI Table1)**


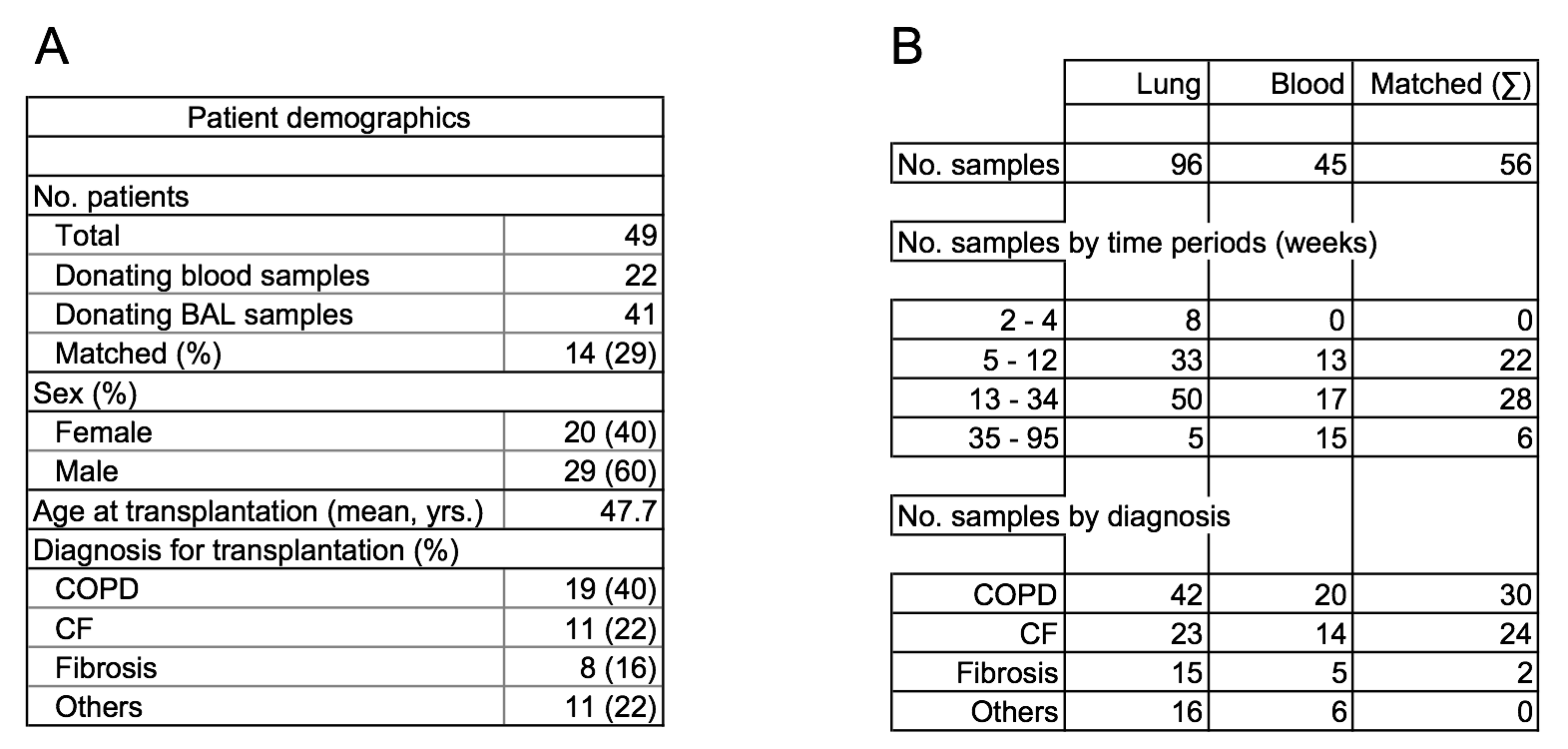


**SI Table1 Description of patient demographics (A) and samples (B) used in this study.**

**(SI Table 2)**

**
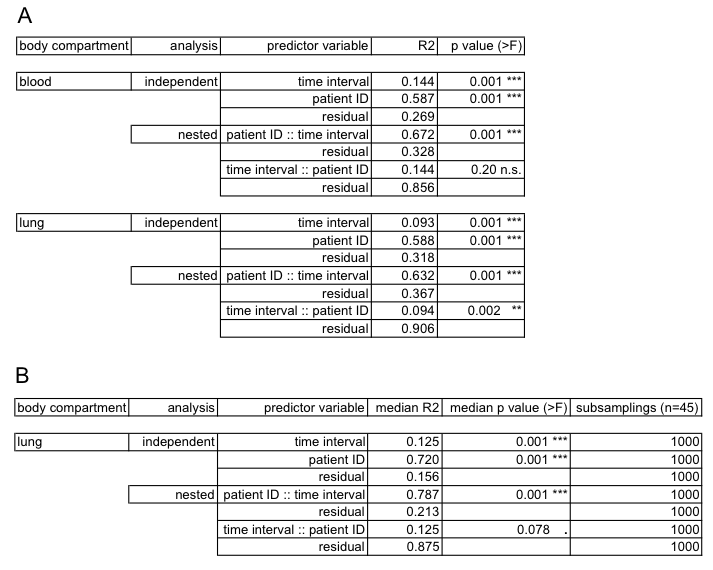
**

**SI Table 2 Data structure and variable hierarchy.**

(**A**) Canonical and nested PERMANOVA variance analysis was performed to determine the importance and hierarchy of metavariables in explaining viral abundances in lung and blood. Time after transplantation was log transformed, categorized by time interval and entered into the model as categorical variable, also patient identity entered as factor. Both variables were tested individually and nested (e.g. time nested in patient ID is denoted as time::patient ID), R^2^ and p values are shown. Sample numbers: n_L_=96, n_P_=45. (**B**) Random subsampling of lung samples to eliminate potential sample number biases between lung and blood. 45 out of 96 lung samples were randomly selected 1000 times and subjected to PERMANOVA as in (A). Median values of R^2^ and p values were reported. Sample number: n_L_=45.
